# It's the virus, stupid – part 2

**DOI:** 10.1186/1742-4690-2-78

**Published:** 2005-12-16

**Authors:** Ben Berkhout

**Affiliations:** 1 Department of Human Retrovirology, Academic Medical Center, University of Amsterdam, Amsterdam, The Netherlands

## Abstract

This editorial presents Retrovirology's choice for the best basic science "retrovirus paper of the year 2005".

Chronic HIV-1 infection is characterized by a steady but generally slow loss of CD4^+ ^T cells. A central puzzle of HIV-1 research in the early 90's has been how the virus could cause AIDS, when it infects a trivial number of T cells. It took until 1995 before it was realized that massive virus replication occurs during chronic infection [1]. A model was proposed in which HIV-1 kills healthy cells slightly faster than the human body is able to replenish. David Ho, in a paraphrase of former US president Bill Clinton, said "It's the virus, stupid" to underscore the primary role of the virus in the pathogenesis of AIDS. This finding sets the stage for the implementation of viral load tests in routine diagnostics. The new concept also provided the rationale for attempts to block the furiously replicating virus with antivirals, instead of resolving the disease by modulating the immune system.

Nevertheless, the frequency of infected peripheral CD4^+ ^T cells in the chronic phase of HIV-1 infection is too low (0.01 – 1%) to account for the ongoing depletion of these cells by viral infection. In addition, the mechanism for the massive and rapid loss during the acute phase of infection remains unknown. Recent work with HIV-1 and SIV in the macaque model demonstrated that acute infection is accompanied by a dramatic and selective loss of memory CD4^+ ^T cells predominantly from the mucosal surfaces, but the mechanism underlying this depletion was not clarified. Two 2005 papers in Nature clearly repeated the old message "It's the virus, stupid – part 2". I find that two of the best basic science retrovirus papers of 2005 are the works from Joseph Mattapallil and colleagues and Gingsheng Li and colleagues describing that HIV-1 infects and kills the memory CD4^+^T cells, a T-cell subset responsible for remembering previous infections [2], [3]. This initial assault may determine the outcome of the lengthy battle between SIV-HIV and its host.

Mattapallil et al. used a technique that can detect a single copy of SIV DNA to show that 30–60% of all memory CD4^+ ^T cells were infected within 10 days of viral challenge. Most of these cells in the infected rhesus macaque had disappeared 4 days later. There is an exclusive loss of memory CD4^+^T cells not only from gut-associated mucosal tissues, but also from organized lymph nodes and peripheral blood. Li et al. characterized the activation status of the SIV-producing cells in tissue sections, which turn out to be predominantly resting lymphocytes. This result may be surprising because this virus is known to replicate more efficiently in activated cells, but mucosal lymphocytes are probably better described as "recently activated". Mattapallil suggests that the high rate of infection of these cells is a sufficient mechanism to account for their loss during acute infection; no bystander mechanisms need to be invoked. But Li suggests that indirect mechanisms also contribute to T-cell death in acute infection. Recent studies have provided compelling evidence that the rapid and profound memory T cell depletion is not a peculiarity of the SIV-macaque model, but is also seen in the gut of acutely infected patients. Much remains to be learned on how the substantial depletion of memory CD4^+ ^T cells during acute infection influences the gradual depletion of CD4^+ ^T cells during the chronic phase, and how the removal of CCR5-positive memory cells relates to the coreceptor switch towards CXCR4 that occurs in some patients.

The two papers clearly demonstrate that our understanding of the pathogenesis of AIDS has been biased by reliance on examination of peripheral blood, which may provide a limited and perhaps even misleading view. The papers also have implications for antiretroviral treatment and the development of vaccines. For instance, mucosal immunity will be essential for designing an effective AIDS vaccine. Early intervention drug therapy and vaccines should ideally prevent the massive destruction of the CD4^+^memory T cell compartment. Countermeasures should be developed to attack the virus at or shortly after transmission. The fight with antivirals or microbicides should be targeted in the cervico-vaginal tissues, the predominant portal of viral entry in the HIV-1 pandemic. It seems particularly important that vaccines should block HIV-1 before the initial burst of viral replication in mucosal lymphocytes. The best way to prevent this is by a vaccine that induces potent neutralizing antibodies, but that is easier said than done.

The new insights may also be relevant for studies that focus on biological differences between the different HIV-1 subtypes. A major determinant of subtype-specific differences in host cell tropism and possibly pathogenesis is encoded by the long terminal repeat (LTR) promoter of HIV-1 [4]. Mireille Centlivre and colleagues inserted the HIV-1 subtype LTRs in SIV and followed viral dissemination in macaque body compartments [5]. In direct virus competition experiments, subtype C replicated preferentially in the gut-associated lymphoid tissue and subtype B was found predominantly in peripheral blood mononuclear cells during acute infection. The different chemokine/interleukin environments are likely to differentially influence the subtype LTR promoters that encode distinct transcription factor binding sites. For instance, the gut-associated lymphoid tissue is an IL-7 rich environment, which specifically activates subtype C replication through enhancement of LTR transcription [6]. Such differences in the pathogenesis of acute HIV-SIV infection may translate into differential disease progression.
